# Assessment of pulmonary veins after atrio-pericardial anastomosis by cardiovascular magnetic resonance

**DOI:** 10.1186/1532-429X-13-72

**Published:** 2011-11-21

**Authors:** Steven C Greenway, Shi-Joon Yoo, Giedrius Baliulis, Christopher Caldarone, John Coles, Lars Grosse-Wortmann

**Affiliations:** 1 Division of Cardiology, The Hospital for Sick Children, University of Toronto, Ontario, M5G 1X8, Canada; 2 Department of Diagnostic Imaging, The Hospital for Sick Children, University of Toronto, Ontario, M5G 1X8, Canada; 3 Division of Cardiovascular Surgery, The Hospital for Sick Children, University of Toronto, Ontario, M5G 1X8, Canada

## Abstract

**Background:**

The atrio-pericardial anastomosis (APA) uses a pericardial pouch to create a large communication between the left atrium and the pulmonary venous contributaries in order to avoid direct suturing of the pulmonary veins during the repair of congenital cardiac malformations. Post-operative imaging is routinely performed by echocardiography but Cardiovascular Magnetic Resonance (CMR) offers excellent anatomical imaging and quantitative information about pulmonary blood flow. We sought to compare the diagnostic value of echocardiography and CMR for assessing pulmonary vein anatomy after the APA.

**Methods:**

This retrospective study evaluated all consecutive patients between October 1998 and January 2010 after either a primary or secondary APA followed by post-repair CMR.

**Results:**

Of 103 patients who had an APA, 31 patients had an analyzable CMR study. The average time to CMR was 24.6 ± 32.5 months post-repair. Echocardiographic findings were confirmed by CMR in 12 patients. There was incomplete imaging by echocardiography in 7 patients and underestimation of pulmonary vein restenosis in 12, when compared to CMR. In total, 19/31 patients (61%) from our cohort had significant stenosis following the APA as assessed by CMR. Our data suggest that at least 18% (19/103) of all patients had significant obstruction post-repair.

**Conclusions:**

Echocardiography incompletely imaged or underestimated the severity of obstruction in patients compared with CMR. Pulmonary vein stenosis remains a sizable complication after repair, even using the APA.

## Background

Pulmonary vein (PV) stenosis carries a poor prognosis due to the difficulty of primary surgical repair and the subsequent risk of recurrent obstruction [1-6]. Pulmonary vein stenosis occurs as a complication in a significant fraction of patients post-repair [7], [8].

An alternative to the "classical" operation for PV stenosis, where the PVs are directly anastomosed to the left atrium (LA), is the so-called "sutureless repair" or atrio-pericardial anastomosis (APA) which creates a large communication between the LA and the PVs using a pericardial pouch while avoiding direct suturing of the divided edges of the veins [4,5,9-11]. It was hoped that avoiding direct surgical trauma to the veins would reduce the incidence of post-operative obstruction. Since it was first introduced for isolated PV stenosis, the use of the APA has been extended to the correction of partial and total anomalous pulmonary venous connections and short- and medium-term outcomes have been encouraging [4,12-14].

The dominant imaging modality in pediatric congenital heart disease is echocardiography but it may not be the optimal technique for visualization and characterization of post-operative PV stenosis. Furthermore, isolated stenosis of one PV is often clinically silent. Cardiovascular Magnetic Resonance (CMR) allows a comprehensive assessment of PV anatomy and function and is able to quantify blood flow through the pulmonary veins and pulmonary arteries to provide sensitive detection of pre- and post-repair PV pathology [15-22].

Based on our previous work, we hypothesized that CMR is superior to conventional transthoracic echocardiography for the detection of PV stenosis post-APA [15,17,18]. The primary aim of this study was to compare the performance of echocardiography and CMR in this surgical cohort. A secondary aim was to examine patient outcome post-APA by CMR.

## Methods

### Patient population

Following approval from our institution's ethics review board, all patients who underwent either primary or secondary APA between October 1998 and January 2010 were identified from the surgical database. Of the 103 patients who had undergone APA, 31 patients had had a CMR that included evaluation of the pulmonary veins post-APA.

Clinical, demographic and surgical information, including type of PV anomaly, associated congenital heart defects (CHDs), age at time of surgery and age at CMR, were collected for all patients. Information on the indications for CMR and whether or not there was suspicion of PV stenosis preceding CMR based on clinical, radiographic or echocardiographic examination was extracted from the patients' medical records.

### CMR imaging

Examinations prior to 2007 were performed on a 1.5 T General Electric magnet ("Signa CV/*I*", General Electric Medical Systems, Milwaukee, WI). From 2007 onwards, a 1.5 T Siemens scanner ("Avanto", Siemens Medical Solutions, Erlangen, Germany) was used. The protocol included static white blood localizer views in three orthogonal body planes, volumetric assessments of both ventricles, phase-contrast flow velocity mapping of the pulmonary arteries and veins, and contrast-enhanced MR angiography. The imaging parameters are described in detail elsewhere [18,19,21]. Ventricular volumes and flow volumes were calculated on a commercially available workstation (MEDIS Medical Imaging Systems, Leiden, The Netherlands). The three-dimensional angiographic datasets were reviewed and reformatted using a commercially available off-line workstation ("Advantage Windows 3.0", GE Medical Systems or "Leonardo", Siemens Medical Solutions).

The average patient age at the time of CMR was 2.7 ± 3.6 years (range 6 days to 14 years) and the average patient weight was 11.4 ± 10.7 kg (range 3 to 50 kg). At our institution patients less than 6 years of age typically are studied under general anesthetic although a "feed-and-sleep" approach has been used successfully in neonates and young infants in order to avoid this.[23]

In a retrospective review of each CMR examination by one of two experienced readers (SJY and LGW), each PV was analyzed for the following features: luminal PV morphology, blood flow velocity, volume and flow curve pattern in each PV, volume and flow curve pattern in the right and left branch pulmonary arteries, ventricular ejection fractions and presence of right ventricular hypertrophy as well as septal flattening. The diagnosis of significant PV obstruction was made if two or more of the following findings were present: discrete PV stenosis and/or severe hypoplasia, loss of phasic flow in the PV, redistribution of blood flow to the contralateral lung and/or to the uninvolved lobes on the ipsilateral side, and evidence of isolated pulmonary hypertension in the flow pattern of the ipsilateral branch pulmonary artery. In borderline cases, a consensus between the two CMR readers was obtained. The earliest CMR study in this cohort was performed in 2001.

## Results

Of the 103 patients who had undergone APA between October 1998 and January 2010 there were 31 patients (30%) who had also had a CMR post-APA that included evaluation of the pulmonary veins.

In our cohort of 31 patients there were 13 individuals (42%) who had primary isolated PV disease without any associated CHDs or whose heart defects did not require surgical repair, e.g. patent foramen ovale, atrial septal defect and/or a persistent arterial duct. The remaining 18 patients had significant other heart defects in addition to the PV abnormalities. Right isomerism of the atrial appendages and atrioventricular septal defect were present in eight patients, four patients had hypoplastic left heart syndrome, two patients had Scimitar syndrome and four patients had either persistent arterial trunk, tricuspid atresia or a ventricular septal defect associated with other cardiac anomalies.

PV anomalies included 19 cases of total anomalous pulmonary venous connection (TAPVC, including supracardiac (n = 7), cardiac (n = 5) and infracardiac (n = 7) types) and 12 patients with PV stenosis, either unilateral (n = 9) or bilateral (n = 3).

The majority of patients underwent a primary APA (27/31, 87%) reflecting the recently increased use of the APA at our institution, but there were four patients who developed stenosis following their initial classical PV repair and the APA surgically relieved this obstruction. These patients underwent re-operation as early as 6 days to as late as 3 years after their first surgery. These four patients were the only ones who underwent additional PV intervention and their CMR was performed after the APA re-operation. On average, all 31 patients underwent CMR 24.6 ± 32.5 months (mean ± standard deviation) post-APA.

In the total cohort of 31 patients with CMR after APA, PV stenosis was suspected in 17 patients (55%) prior to CMR, by clinical examination (n = 6, including decreased oxygen saturation, signs of respiratory distress, or hemoptysis), increased interstitial markings on chest radiography (n = 3), echocardiography (n = 13, 76%), cardiac catheterization (n = 2, stenosis or decreased PV return) or a combination of the above (n = 7). In the patients with an abnormal physical exam or chest radiograph all, except one, had an abnormal echocardiogram.

CMR confirmed the echocardiographic findings in 12/31 patients. However, echocardiography incompletely imaged the PVs in seven patients (22%) and, in 12 patients (39%), the CMR findings were worse than expected based on the echocardiographic findings (Table 1). Therefore, overall, 61% (19/31) of patients had important post-APA PV stenosis that was incompletely identified by echocardiography. In those patients where the severity of PV restenosis was underestimated by echocardiography there was no obvious anatomical pattern with regards to which PV was affected. Echocardiography missed isolated stenosis of single PVs and did not detect complete obliteration of PVs or obstruction of multiple PVs in four patients. On average, CMR was performed 209 ± 472 days after echocardiography (range 0 to 2053 days) with no significant difference (p = 0.79 by Student's t-test) between the group of patients with (233 ± 580 days, range 0 to 2053 days) or without (177 ± 254 days, range 0 to 658 days) PV stenosis missed by echocardiography.

**Table 1:** Findings for 12/31 patients where echocardiography underestimated the severity of pulmonary vein restenosis compared with CMR.

Patient	ECHO findings	CMR findings
2	Large unobstructed RPVs with laminar flow.	Severe discrete stenosis at junction of RLPV to LA.

6	Laminar flow in LUPV.	LPVs compressed between atria and descending aorta.

9	Long segment narrowing of LUPV with mild flow acceleration. Normal low velocity phasic flow in all 3 remaining PVs.	No LPVs are definitely identified on angiography.

13	4 PVs with laminar color flow and low velocity biphasic flow.	LUPV shows stenosis at its connection to the confluence.

17	Biphasic flow in all PVs.	Hypoplasia of the RUPV and LLPV. Some discrete narrowing of RLPV at entrance to LA.

18	Laminar flow in RPVs. Turbulent but phasic flow in LPVs.	Complete occlusion of RUPV. LPVs have significant focal area of narrowing at LA junction.

24	Flow acceleration seen in LLPV.	LUPVs remain diffusely stenotic... the entry of the LLPV into the LA is severely stenotic. Mild narrowing of RLPV prior to its confluence with the more inferior vein.

25	Stenosis of LLPV to LA. Laminar flow in LUPV and RPVs.	LUPV and LLPV are obstructed.

26	RLPV to IVC. RUPV to LA.	Mild constriction and kinking of RUPV as it connects to LA. Mild stenosis of the RLPV as it connects to the IVC.

28	Increased flow velocity in LPVs. Unobstructed RPVs.	Complete obstruction/atresia of the LPVs. Moderate compression of the common RPV.

29	RPVs have mild flow acceleration with low velocity, phasic flow. Increased but phasic flow across LLPV.	Only lower PVs connected to the LA. RLPV is intact without any stenosis. LLPV has discrete (~50%) stenosis.

30	Laminar color flow seen in 4 PVs. LUPV not seen more distally but laminar color flow seen entering the LA.	LUPV is small and presents a severe discrete stenosis at its junction to the LA.

IVC, inferior vena cava; LA, left atrium; LLPV, left lower pulmonary vein; LPVs, left-sided pulmonary veins; LUPV, left upper pulmonary vein; PV, pulmonary vein; RLPV, right lower pulmonary vein; RPVs, right-sided pulmonary veins; RUPV, right upper pulmonary vein.

In general, estimates of pulmonary blood flow calculated from CMR reflected the obstruction seen by CMR angiography: PV stenosis by angiography led to a decreased perfusion to the ipsilateral lung (data not shown). However, in a patient with severe discrete stenosis at the junction of the right lower PV to LA, equal blood flow was maintained to both lungs presumably due to the ability of the right upper PV to compensate. Another patient had hypoplasia of the right upper PV and the left lower PV and therefore the distribution of pulmonary blood flow was balanced to the right and left lungs and could not be used as a marker of PV obstruction in this case.

## Discussion

Our findings suggest that CMR is superior in the detection of PV stenosis post-APA when compared to echocardiography, chest radiographs, clinical picture or the combination of these. This is supported by our previous work documenting the improved visualization of the normal [21] and surgically repaired [18] PVs by CMR compared to echocardiography.

In accordance with our conclusion, a recent multi-centre study in 58 patients with PV stenosis found that 26% of stenoses were not detected by echocardiography but were subsequently diagnosed by angiography or at the time of surgery [2]. The authors speculate that these cases of narrowing were missed by echocardiography because Doppler velocities may not be accelerated and "flattened" as signs of obstruction in the presence of redistribution of blood flow from the respective lung segments.

In a study by Oh et al., multidetector CT and echocardiography were compared in their ability to identify the drainage site of the common PV in TAPVC as well as to detect stenosis of the PVs and vertical vein [24]. Compared to their gold standard, direct detection at the time of surgery, the investigators found that echocardiography had a sensitivity of only 71% for identifying stenosis of the vertical vein in comparison to CT which had a sensitivity of 100% [24]. These findings strongly advocate for cross-sectional imaging for the diagnosis of PV stenosis. Magnetic resonance imaging has the obvious additional benefits of supplying flow information and not exposing the developing child to ionizing radiation.

"Classical" repair of TAPVC is complicated by late PV obstruction in nearly 8% of patients [5], although this may be an underestimate since CMR was not used. Indeed, in a recent review of their experience, Kelle et al. report an approximately 20% stenosis rate following TAPVC repair [1]. It was hoped that the APA, by avoiding direct vessel trauma from suturing, would have a lower incidence of post-repair PV stenosis. However, our data suggests that, despite a change in surgical technique, there remains a significant risk of PV narrowing following the APA. At least 18% of all patients (19/103) had post-APA PV stenosis. Three patients in our cohort were not clinically suspected to have PV stenosis prior to CMR and there may be additional post-APA patients with asymptomatic PV restenosis who were not evaluated by CMR. Therefore, we conclude that at least 18%, but likely more, of post-APA patients have post-repair obstruction.

In our study, two out of four patients treated by APA for post-repair stenosis developed recurrent PV stenosis, highlighting the difficulty of repair in this patient subgroup. Others have identified patients with a functionally single ventricle and/or heterotaxy as being at significantly higher risk for reoperation [1] but, in our study, only five of the 19 patients with restenosis had a single ventricle or heterotaxy so these factors did not appear to be obvious complicating factors, although the relatively small number of patients precluded a significant regression analysis of our data.

### Study limitations

A limitation of our study was the fact that only 31 out of 103 patients who underwent APA were studied with CMR. The exact reasons for the limited number of patients studied are not available but most probably are primarily related to clinician preference. In some of the studies there was a considerable time span between the echocardiographic and CMR imaging of the pulmonary veins. Although PV obstruction is a dynamic process there was not a significant difference in imaging interval between the group of patients with and without differences in PV stenosis. As even severe pulmonary venous obstruction may remain clinically silent for a long time, the true prevalence of PV stenosis following APA cannot be determined from our retrospective study, but is at least 18% and likely higher. Another limitation of our retrospective study was the lack of hemodynamic data to correlate with the CMR findings. The inclusion of this would control for a potential overestimation of PV stenosis due to thresholding of volume rendering in CMR angiography. These limitations clearly demonstrate the need for a prospective study targeting all patients who have undergone APA, ideally in a comparison with the "classic" technique.

**Figure 1:**
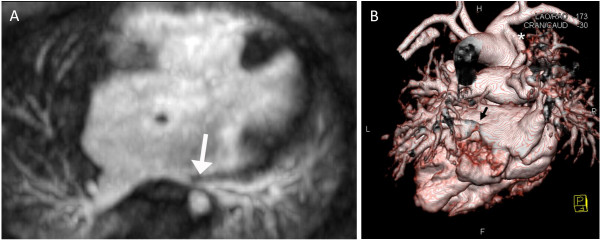
**Nine year-old male with right isomerism of the atrial appendages, bilateral superior venae cavae, atrioventricular septal defect, pulmonary atresia and total anomalous pulmonary venous connections**. CMR was performed 3 months after the APA repair. A) Axial image showing narrowing of the left-sided common pulmonary venous confluence due to compression between the left-sided atrium and the aorta (arrow). B) Volume-rendered image with the descending aorta removed showing stenosis of the left lower pulmonary vein (arrow) and the modified Blalock-Taussig shunt (*). F, feet; H, head; L, left; R, right.

**Figure 2:**
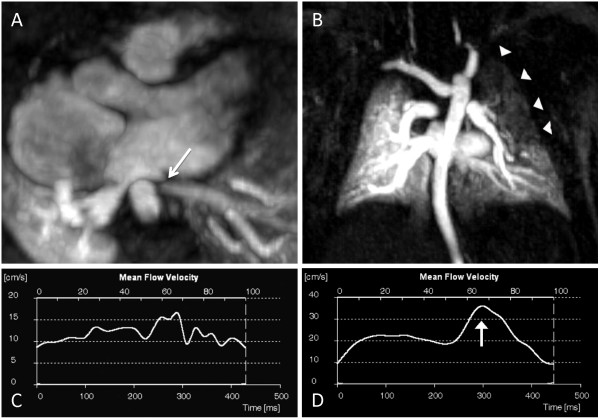
**Premature infant who underwent CMR at 8 weeks corrected gestational age weighing 3.0 kg**. The original diagnosis was total anomalous pulmonary venous connections (cardiac type) repaired by the APA. A) Axial image shows severe narrowing of the left upper PV post-operatively (arrow). B) Maximum intensity projection of the magnetic resonance angiography dataset demonstrates delayed filling of the left lung, particularly of the upper lobe (arrowheads). The left upper PV is not shown on this first pass angiographic acquisition, but filled during the second acquisition 12 seconds later. C) Time-velocity flow curve in the left lower PV reveals loss of phasic flow at a relatively low velocity (proximal to the narrowing). D) Normal post-operative flow pattern in the common right PV in the same patient, with two distinct peaks, the greater one during diastole (arrow).. Only 27% of pulmonary blood flow went to the left lung. Note the different velocity scales for the left (C) and right (D) PV flows.

## Conclusions

In conclusion, we have shown that CMR is a sensitive imaging modality that offers enhanced detection of obstructive pulmonary vein lesions in comparison to echocardiography and other clinical tests. Our data also suggests that, in the long-term, the APA is complicated by a high rate of PV restenosis, which may be comparable to rates seen after classical repair. In our opinion and experience, surveillance scans with CMR are clinically indicated as we have shown that other screening tools, including clinical examination and echocardiography, lack sufficient sensitivity to detect significant stenosis or restenosis. Furthermore, any abnormality of the PVs seen on echocardiography should be followed-up with CMR for a more detailed assessment of the PV anatomy and severity of obstruction.

## List of abbreviations used in the text

PV: pulmonary vein; LA: left atrium; APA: atrio-pericardial anastomosis; CMR: Cardiovascular Magnetic Resonance; CHDs: congenital heart defects; TAPVC: total anomalous pulmonary venous connections.

## Competing interests

The authors declare that they have no competing interests.

## Authors' contributions

SCG, LGW and SJY performed the analysis. All authors contributed to the study design, acquisition and interpretation of the data and the writing of the manuscript. All authors have read and approved the final manuscript.
